# ICIs-Related Cardiotoxicity in Different Types of Cancer

**DOI:** 10.3390/jcdd9070203

**Published:** 2022-06-28

**Authors:** Mei Dong, Ting Yu, Zhenzhen Zhang, Jing Zhang, Rujian Wang, Gary Tse, Tong Liu, Lin Zhong

**Affiliations:** 1 Department of Cardiology, The Affiliated Yantai Yuhuangding Hospital of Qingdao University, Yantai 264000, China; dongmei0212@126.com (M.D.); m18663817808@163.com (Z.Z.); zhangjingfriend@126.com (J.Z.); 2 Medical College, Qingdao University, Qingdao 266003, China; 13061487578@163.com; 3 Department of Oncology, The Affiliated Yantai Yuhuangding Hospital of Qingdao University, Yantai 264000, China; w15066122363@126.com; 4 Tianjin Key Laboratory of Ionic-Molecular Function of Cardiovascular Disease, Department of Cardiology, Tianjin Institute of Cardiology, Second Hospital of Tianjin Medical University, Tianjin 300070, China; gary.tse@doctors.org.uk; 5 Kent and Medway Medical School, Canterbury CT2 7FS, UK

**Keywords:** immune checkpoint inhibitors, cardiotoxicity, cardio-oncology, cancer-type-specific

## Abstract

Immune checkpoint inhibitors (ICIs) are rapidly developing immunotherapy cancer drugs that have prolonged patient survival. However, ICIs-related cardiotoxicity has been recognized as a rare, but fatal, consequence. Although there has been extensive research based on different types of ICIs, these studies have not indicated whether cardiotoxicity is specific to a type of cancer. Therefore, we conducted a systematic review to analyze a variety of ICIs-related cardiotoxicity, focusing on different types of cancer. We found that the incidence of ICIs-related cardiac adverse events (CAEs) and common cardiotoxic manifestations vary with cancer type. This inspired us to explore the underlying mechanisms to formulate targeted clinical strategies for maintaining the cardiovascular health of cancer patients.

## 1. Introduction

**Figure 1:**
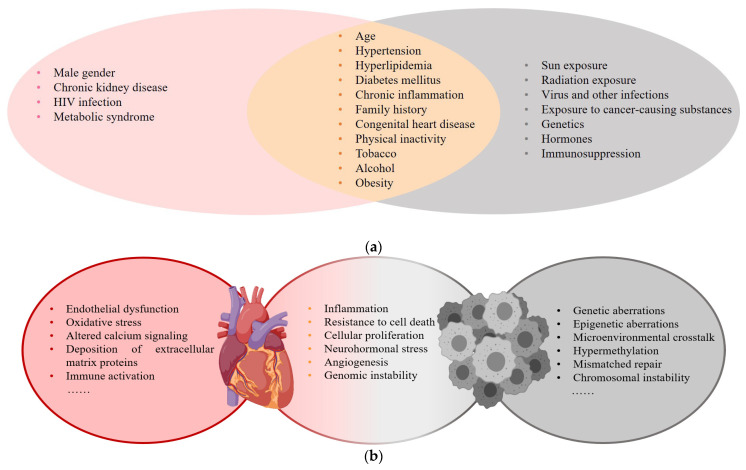
(**a**) Risk factors for CVD and cancer; (**b**) Common pathophysiologic processes of CVD and cancer.

Cardiovascular disease (CVD) and cancer are global health issues with high morbidity and mortality [1], and numerous published studies suggest that there is an overlap in epidemiology, risk factors, and pathophysiologic processes (Figure 1) [1,2,3,4,5].

With the widespread application of anticancer drugs, the survival of patients has significantly improved, but the related cardiotoxicity affects long-term therapeutic outcomes, and this has attracted considerable attention. Immune checkpoint inhibitors (ICIs), antibodies that target the checkpoints in immune cells, work to activate inhibited T-cells and other cells of the innate and adaptive arms, resulting in the robust activation of the immune system and productive antitumor immune responses. This new type of immunotherapy drug has significantly improved the survival of cancer patients [6,7,8]. ICIs have been widely used in the treatment of melanomas, non-small cell lung cancer (NSCLC), advanced renal cell carcinomas (RCCs), urothelial carcinomas, hepatocellular carcinomas (HCCs), and hematological malignancies [7,9,10,11,12]. However, their use is associated with adverse side effects involving different organs [13,14]. ICIs-related cardiotoxicity, which may develop even without a history of significant cardiac risk factors, includes myocarditis, pericarditis, heart failure, arrhythmias, and vasculitis [15]. In reported cases of adverse ICIs-related events, 6.2% were cardiac adverse events (CAEs), which can be the main determinants of quality of life and increased mortality [3,16,17]. Recent cohort data from a large healthcare network suggested that the most common CAEs were arrhythmia (9.3%) and myocarditis (2.1%) [18]. Cardiotoxicity associated with ICIs is known for its vast array of clinical presentations, which makes it unfavorable for an early diagnosis [19,20]. To date, there has been little agreement on the incidence or specific mechanisms of ICIs-related cardiotoxicity in different types of cancer. We hypothesize that ICIs may exhibit cancer-type-specific cardiotoxicity.

## 2. Methods

We systematically reviewed articles published up to 28 February 2022 in PubMed, Web of Science, and Google Scholar databases without any language restrictions. The keywords included “PD-1”, “PD-L1”, “CTLA-4”, “LAG-3”, “nivolumab (anti-PD-1 antibody)”, “pembrolizumab (anti-PD-1 antibody)”, “atezolizumab (anti-PD-L1 anti-body)”, “durvalumab (anti-PD-L1 antibody)”, “ipilimumab (anti-CTLA-4 antibody)” (with their chemical names and brand names), “cancer”, “tumor”, “carcinoma”, “neoplasm”, “malignancy”, “adverse events”, “complications”, and “cardiotoxicity”. The inclusion criteria of papers were (1) retrospective and prospective studies, case reports, meta-analysis, reviews involving PD-1, PD-L1, CTLA-4 and LAG-3 inhibitors for all cancers; (2) data on the rates of any ICIs-related adverse events associated with cardiac disorders. The exclusion criteria were as follows: (1) patients treated with anthracyclines (such as doxorubicin, daunorubicin, or idarubicin); (2) patients treated with tyrosine inhibitor kinase drugs, T-cell activated cells, activated dendritic cells, stem cell transplantation, or other antibodies; and (3) patients treated with ICIs with concomitant vaccines. A total of 549 papers were found of which 102 were kept for this review. Eventually, more than 40 clinical trials and case reports of 14 different cancers were collected.

## 3. Cardiotoxicity in Different Types of Cancer

### 3.1. Melanoma

In 16 studies, 24 of 6710 patients on ICIs [21,22,23,24,25,26,27,28,29,30,31,32,33,34,35,36] developed CAEs. This corresponded with an incidence of 0.20–4.93% in which grade 3–5 CAEs accounted for 41.7%. Commonly encountered cardiotoxicities included hypertension (50%), hypotension (16.7%), and myocarditis (8.3%). Treatment-related hypertension was linked to the application of lambrolizumab (58.3%) (PD-1). Nivolumab may have had a correlation with ICIs-related hypotension. Patients treated with a higher dose of ipilimumab, particularly 10 mg/kg × 4 doses/3 weeks, were more prone to fatal adverse events such as cardiac arrest (Table 1).

### 3.2. Lung Cancer

A total of 11 studies [37,38,39,40,41,42,43,44,45,46,47] included 5404 patients on ICIs, and 101 developed CAEs for an incidence of 0.15–37.78% in which grade 3–5 CAEs accounted for 55.4%. Commonly encountered cardiotoxicities included arrhythmia (32.7%), cardiac-related chest pain (24.8%), elevated cTnI or myocarditis (23.8%), cardiomyopathy (20.8%), pericardial disease (11.9%), and acute coronary syndrome (10.9%). One study indicated that major adverse cardiovascular events (MACEs) were dose-independent of nivolumab and pembrolizumab in lung cancer patients [37]. Those treated with a higher dose of durvalumab, particularly 10 mg/kg × 4 doses/2 weeks, were more prone to fatal adverse events such as a cardiac arrest and cardiogenic shock [41]. One patient treated with pembrolizumab at 10 mg/kg for 3 weeks underwent a myocardial infarction, which led to death (Table 2) [43].

### 3.3. Renal Cell Carcinoma

In seven studies [48,49,50,51,52,53,54] comprising 1971 patients with renal cell carcinomas on ICIs, 14 developed CAEs with an incidence of 0.20–2.19% in which grade 3–5 CAEs accounted for 35.7%. Commonly encountered cardiotoxicities included hypertension (85.7%) and myocarditis (7.1%). Treatment-related hypertension was linked to a nivolumab plus ipilimumab therapy (100%). Compared with melanomas and lung cancer, the ICI therapy caused mild cardiotoxicity in renal cell carcinomas. Fatal CAEs were not found (Table 3).

### 3.4. Urothelial Carcinoma

In Seven studies [55,56,57,58,59,60,61] 111 of 2550 patients with urothelial carcinomas on ICIs developed CAEs with an incidence of 0.22–10.60% in which grade 3–5 CAEs accounted for 52.3%. Commonly encountered cardiotoxicities included hypertension (28.8%), arrhythmia (14.4%) and hypotension (6.3%). The fluctuation of blood pressure was linked to treatment with atezolizumab. Hypertension was observed in 21 patients and hypotension was observed in 7 after application of atezolizumab. Patients treated with 200 mg pembrolizumab for 3 weeks (maximum 35 cycles) or at 1200 mg every three weeks were more prone to fatal adverse events such as a cardiac arrest (Table 4).

**Table 1:** Cardiotoxicity in melanoma.

Author, Year	Study Type	Phase	Sample Size	Drug	Dose and Frequency	Non-CAE	CAE	Manifestation	3–5 Grade CAE
Omid Hamid et al., 2017 [21]	Prospective study	II	528 (178 vs. 179 vs. 171)	Pembrolizumab vs. Pembrolizumab vs. chemotherapy	2 mg/kg/3 weeks vs. 10 mg/kg/3 weeks vs. standard dose	528	0	0	0
Caroline Robert et al., 2014 [22]	Prospective study	III	418 (210 vs. 208)	Nivolumab vs. Dacarbazine	3 mg/kg/2 weeks vs. standard dose	308 (153 vs. 155)	5	Hypotension 1 vs. 4	0
Jeffrey S Weber et al., 2015 [23]	Prospective study	III	370 (268 vs. 102)	Nivolumab vs. ICC (Dacarbazine al)	3 mg/kg/2 weeks vs. standard dose	362 (181 vs. 81)	0	0	0
Paolo A Ascierto et al., 2017 [24]	Prospective study	III	726 (364 vs. 362)	Ipilimumab	10 mg/kg/4 doses/3 weeks vs. 3 mg/kg/4 doses/3 weeks	514 (286 vs. 228)	3	Hypertension 1 vs. 0; Heart arrest 1 vs. 0; Pericarditis 1 vs. 0	3
F Stephen Hodi et al., 2016 [25]	Prospective study	II	142 (95 vs. 47)	Nivolumab + Ipilimumab vs. Ipilimumab + placebo	1 mg/kg + 3 mg/kg/4 doses/3 weeks vs. 3 mg/kg + placebo/4 doses/3 weeks	140 (94 vs. 46)	7	Hypotension 3 vs. 0; Ventricular arrhythmia 1 vs. 0; Ventricular tachycardia 1 vs. 0; Atrial fibrillation 1 vs. 0; Myocardial infarction 1 vs. 0	5
Caroline Robert et al., 2015 [26]	Prospective study	III	834 (278 vs. 277 vs. 256)	Pembrolizumab vs. Pembrolizumab vs. Ipilimumab	10 mg/kg/2 weeks/doses vs. 10 mg/kg/3 weeks/ doses vs. 3 mg/kg/3 weeks/4 doses	610 (221 vs. 202 vs. 187)	4	Hypertension 3 vs. 1 vs. 0	2
J. Weber, M. et al., 2017 [27]	Prospective study	III	906 (453 vs. 453)	Nivolumab vs. Ipilimumab	3 mg/kg/4 doses/2 weeks vs. 10 mg/kg/4 doses/3 weeks	884 (438 vs. 446)	0	0	0
J.D. Wolchok et al., 2017 [28]	Prospective study	III	937 (313 vs. 313 vs. 311)	Nivolumab + Ipilimumab vs. Nivolumab + p vs. Ipilimumab + p p(placebo)	1 mg/kg+3 mg/kg /3 weeks/4 doses vs. 3 mg/kg/2 weeks + placebo vs. 3 mg/kg/3 weeks/4 doses + placebo	847 (300 vs. 279 vs. 268)	0	0	0
Jedd D Wolchok et al., 2010 [29]	Prospective study	II	217 (73 vs. 72 vs. 72)	Ipilimumab	10 mg/kg vs. 3 mg/kg vs. 0.3 mg/kg/3 weeks/4 doses	115 (50 vs. 46 vs. 19)	0	0	0
Ines Pires da Silva et al., 2021 [30]	Retrospective study	NR (Not Reported)	355 (193 vs. 162)	Ipilimumab + Nivolumab/Pembrolizumab/Atezolizumab vs. Ipilimumab	3 mg/kg/3 weeks/4 doses + standard dose vs. 3 mg/kg/3 weeks/4 doses	287 (163 vs. 124)	1 (0 vs. 1)	Myocarditis 0 vs. 1	1
Patrick Schöffski et al., 2022 [31]	Retrospective study	I/II	255 (134 vs. 121)	LAG-3 inhibitor Ieramilimab vs. Ieramilimab + Spartalizumab	Ieramilimab (escalating 1–15 mg/kg)/2 weeks or once/4 weeks vs. Ieramilimab + Spartalizumab q2w or q3w or q4w or Ieramilimab q2w + Spartalizumab q4w	159 (75 vs. 84)	0	0	0
Alexander M.M. et al., 2020 [32]	Prospective study	III	1011 (509 vs. 502)	Pembrolizumab vs. placebo	200 mg/3 weeks for 18 doses	235 (190 vs. 45)	1 (1 vs. 0)	Myocarditis 1 vs. 0	NR
Omid Hamid et al., 2013 [33]	Prospective study	I	135 (57 vs. 56 vs. 22)	Lambrolizumab	10 mg/kg/2 weeks vs. 10 mg/kg/3 weeks vs. 2 mg/kg/3 weeks	132 (55 vs. 55 vs. 22)	7 (2 vs. 4 vs. 1)	Hypertension (2 vs. 4 vs. 1)	NR
Margaret K. et al., 2018 [34]	Retrospective study	I	94 (53 vs. 41)	Ipilimumab + Nivolumab Nivolumab (Niv) Ipilimumab (Ipi)	Niv+Ipi(escalating doses)/3 weeks for four doses, followed by Niv 3 weeks for four doses, then Niv + Ipi/12 weeks for eight doses vs. Niv 1 mg/kg + Ipi 3 mg/kg/3 weeks for 4 doses, followed by Niv 3 mg/kg/2 weeks	87	0	0	0
Ulrich Keilholz et al., 2019 [35]	Prospective study	I	51	Avelumab	10 mg/kg for one-hour intravenous infusion/2 weeks	39	0	0	0
Hussein A et al., 2022 [36]	Retrospective study	II-III	714 (355 vs. 359)	Relatlimab + Nivolumab vs. Nivolumab	Relatlimab 160 mg + Nivolumab 480 mg vs. Nivolumab 480 mg	504 (288 vs. 216)	0	0	0

The severity of adverse events was graded according to the National Cancer Institute Common Terminology Criteria for Adverse Events (CTCAE) Version 5.0. Grade 3: severe or medically significant but not immediately life-threatening; hospitalization or prolongation of hospitalization indicated; disabling; limiting self-care activities of daily living. Grade 4: life-threatening consequences; urgent intervention indicated. Grade 5: Death related to adverse events.

**Table 2:** Cardiotoxicity in lung cancer.

Author, Year	Study Type	Phase	Sample Size	Drug	Dose and Frequency	Non-CAE	CAE	Manifestation	3–5 Grade CAE
Kalyan R et al., 2019 [37]	Retrospective study	NR	252 (117 vs. 135)	Non-ICI vs. ICI (Nivolumab/Pembrolizumab) Nivolumab (Niv) Pembrolizumab (Pem)	Standard dose vs. increasing dose (Niv < 540 mg; 540~1440 mg; > 1440 mg Pem < 600 mg; 600~1707 mg; >1707 mg)	NR	93 (42 vs. 51)	Arrhythmia 31 vs. 25; Cardiac-related chest pain 12 vs. 25; Valvular heart disease 4 vs. 2; Cardiomyopathy 13 vs. 20; Myopericardial disease 11; Pericardial disease 8; Myocarditis 1; Valvular-disease 2; Venous arterial thromboembolic events 8	40 (major CAE)
Scott N et al., 2015 [38]	Prospective study (NSCLC)	I	129 (33 vs. 37 vs. 59)	Nivolumab	1 mg/kg vs. 3 mg/kg vs. 10 mg/kg intravenously/2 weeks in 8-week cycles for up to 96 weeks.	91 (21 vs. 25 vs. 45)	0	0	0
Tony S K Mok et al., 2019 [39]	Prospective study (NSCLC)	III	1251 (636 vs. 615)	Pembrolizumab vs. platinum-based chemotherapy	200 mg/3 weeks for up to 35 cycles vs. platinum-based chemotherapy for four to six cycles.	1112 (515 vs. 597)	1 (1 vs. 0)	Myocarditis 1 vs. 0	1
Achim Rittmeyer et al., 2017 [40]	Prospective study (NSCLC)	III	1187 (609 vs. 578)	Atezolizumab vs. Docetaxel	1200 mg/3 weeks vs. 75 mg/m^2^/3 weeks	886 (390 vs. 496)	0	0	0
S.J. Antonia et al., 2017 [41]	Prospective study (NSCLC)	III	718 (475 vs. 234)	Durvalumab vs. Placebo	10 mg/kg/2 weeks for up to 12 months vs. placebo	421 (301 vs. 120)	26 (21 vs. 5)	ACS 9 vs. 2; Arrhythmia 7 vs. 1; Heart failure 7 vs. 0; Cardiac arrest 2 vs. 1; Cardiogenic shock 1 vs. 0; Cardiomyopathy 1 vs. 0; Myocarditis 0 vs. 1; Pericardial effusion 2 vs. 0	NR
Yuequan Shi et al., 2021 [42]	Observational study (NSCLC/SCLC)	NR	1905 (1162 vs. 743) (598 vs. 455 vs. 273 vs. 176 vs. 125 vs. 81 vs. 62 vs. 34 vs. 23)	ICI (Pembrolizumab/Nivolumab/Camrelizumab/Treprizumab/Tisilizumab/Atezolizumab/Durvalumab/Ipilimumab) only vs. combination therapy	at least one dose	647	22 (22 vs. 0)	Elevated cTnI or myocarditis 22	9
Roy S Herbst et al., 2016 [43]	Prospective study (NSCLC)	II/III	991 (339 vs. 343 vs. 309)	Pembrolizumab vs. Docetaxel	Pem 2 mg/kg, Pem 10 mg/kg vs. Docetaxel 75 mg/m^2^/3 weeks	690 (215 vs. 225 vs. 250)	1 (0 vs. 1 vs. 1)	Myocardial infarction 0 vs. 1 vs. 0; Acute cardiac failure 0 vs. 0 vs. 1	1
Martin Reck et al., 2016 [44]	Prospective study (NSCLC)	III	304 (154 vs. 150)	Pembrolizumab vs. platinum-based chemotherapy	200 mg/3 weeks vs. standard dose	52 (45 vs. 7)	0	0	0
H. Borghaei et al., 2015 [45]	Prospective study (NSCLC)	III	555 (278 vs. 268)	Nivolumab vs. Docetaxel	3 mg/kg/2 weeks vs. 75 mg/m^2^/3 weeks	432 (196 vs. 236)	3 (3 vs. 0)	Cardiac tamponade 1 vs. 0; Pericardial effusion 1 vs. 0 Tachycardia 1 vs. 0	3
Julie Brahmer et al., 2015 [46]	Prospective study (NSCLC)	III	272 (135:137)	Nivolumab vs. Docetaxel	3 mg/kg/2 weeks vs. 75 mg/m^2^/3 weeks.	187 (76 vs. 111)	0	0	0
D.P. Carbone et al., 2017 [47]	Prospective study (NSCLC)	III	530 (267 vs. 263)	Nivolumab vs. Chemotherapy(platinum-based)	3 mg/kg/2 weeks vs. standard dose for six cycles.	431 (188 vs. 243)	2 (2 vs. 0)	Myocardial infarction 1 vs. 0; Pericardial effusion malignant 1 vs. 0	2

**Table 3:** Cardiotoxicity in renal cell carcinoma.

Author, Year	Study Type	Phase	Sample Size	Drug	Dose and Frequency	Non-CAE	CAE	Manifestation	3–5 Grade CAE
Sarah Abou Alaiwi et al., 2019 [48]	Retrospective study	III	499	Anti-PD-1/PD-L1 (Nivolumab/Pembrolizumab/Atezolizumab/Avelumab/Durvalumab)	NR	79	1	Myocarditis 1	1
Emre Yekedüz et al., 2021 [49]	Retrospective study	II/III	173	Nivolumab	Nivolumab 240 mg/2wks	11 (treatment discontinuation)	0	0	0
Robert J Motzer et al., 2018 [50]	Retrospective study	III	1082 (547 vs. 535)	Nivolumab + Ipilimumab vs. sunitinib	3 mg/kg + 1 mg/kg/3 weeks for four doses, followed by Niv 3 mg/kg/2 weeks; or SUN 50 mg orally once daily for 4 weeks (6-week cycle).	273 vs. 305	12 (12 vs. 0)	Hypertension 12 vs. 0	4
Robert J. Motzer et al., 2015 [51]	Prospective study	II	167 (59 vs. 54 vs. 54)	Nivolumab	0.3, 2 or 10 mg/kg intravenously once/3 weeks	47 vs. 45 vs. 49	1 (1 vs. 0 vs. 0)	Cardiac disorder 1 vs. 0 vs. 0	0
Joshua J et al., 2020 [52]	Prospective study	IIIb/IV	97	Nivolumab	240 mg/2 weeks for ≤24 months	68	0	0	0
Robert J. Motzer et al., 2015 [53]	Prospective study	III	406 vs. 397	Nivolumab vs. Everolimus	3 mg/kg intravenously ≥ 60 min/2 weeks vs. 10 mg orally once daily.	319 vs. 349	0	0	0
Ulka Vaishampayan et al., 2019 [54]	Prospective study	I	82 (62 vs. 20) (1Line vs. 2 Line)	Avelumab	10 mg/kg by intravenous Infusion/2 weeks	51 vs. 14	0	0	0

**Table 4:** Cardiotoxicity in urothelial carcinoma.

Author, Year	Study Type	Phase	Sample Size	Drug	Dose and Frequency	Non-CAE	CAE	Manifestation	3–5 Grade CAE
Joaquim Bellmunt et al., 2021 [55]	Prospective study	III	406 vs. 403	Atezolizumab vs. observation group	1200 mg intravenously vs. observation	378 vs. 389	51 (27 vs. 24)	Hypertension 15 vs. 0; Arrythmia 10 vs. 0; Myocardial infarction 1 vs. 0; Cardiac discomfort 2 vs. 0	9
Dingwei Ye et al., 2021 [56]	Retrospective study	II	113	Tislelizumab	200 mg intravenously /3weeks	106 (31 immune - related AEs )	0	0	0
Thomas Powles et al., 2020 [57]	Prospective study	III	345 vs. 340 vs. 313	Durvalumab vs. Durvalumab + Tremelimumab vs. Chemotherapy	1500 mg intravenously/4 weeks vs. Dur + Tre 75 mg intravenously/4 weeks for 4 doses vs. standard dose	193 vs. 254 vs. 282	0	0	0
Padmanee Sharma et al., 2017 [58]	Prospective study	II	270	Nivolumab	3 mg/kg/2weeks	173	1	Cardiovascular failure 1	1
Michiel S. van der Heijden et al., 2021 [59]	Prospective study	III	443 vs. 459	Chemotherapy vs. Atezolizumab	standard dose vs. 1200 mg/3weeks	435 vs. 436	2 (1 vs. 1)	Cardiac arrest 0 vs. 1	1
Jonathan E Rosenberg et al., 2016 [60]	Prospective study	II	315	Atezolizumab	Intravenously given/3weeks	202	13	Hypotension 7; Hypertension 6	5
Thomas Powles et al., 2021 [61]	Prospective study	III	349 vs. 302 vs. 342	Pembrolizumab (Pem)+ chemotherapy vs. Pembrolizumab vs. Chemotherapy	Pem 200 mg/3 weeks for a max of 35 cycles + standard dose vs. Pem only vs. chemo only	NR	98 (40 vs. 29 vs. 29)	Hypertension 8 vs. 3 vs. 2; Atrial fibrillation 4 vs. 2 vs. 2; ACS 4 vs. 2 vs. 3; Cardiac arrest 3 vs. 2 vs. 1 (specific number NR)	42 (18 vs. 14 vs. 10)

### 3.5. Other Types of Cancer

The most commonly encountered ICIs-related type of cardiotoxicity in hematological malignancies was hypertension [62,63,64,65]. In other cancers, such as hepatocellular carcinomas and malignant pleural mesotheliomas, the relevant research did not present many cases [66,67,68,69,70,71]; these were almost all case reports of myocarditis [72,73,74].

## 4. Discussion

A total of 23,090 subjects from more than 40 studies were analyzed and the major findings were (1) ICIs-related CAEs commonly occur in melanomas, lung cancer, urothelial and renal cell carcinomas, and hematological malignancies. The incidence of ICIs-related CAEs ranged from 0.15 to 10%. The most commonly encountered type of cardiotoxicity in melanomas, renal cell carcinomas, and urothelial carcinomas was hypertension, whereas in lung cancer it was arrhythmia. ICIs-related cardiotoxicities for other cancer types appeared mostly in case reports and presented with myocarditis. (2) Among the abovementioned five cancers, the incidence of grade 3–5 ICIs-related CAEs ranged from 35.7 to 55.4%. Compared with RCCs, the other four types had a higher incidence of CAEs, including sudden cardiac arrest. (3) In different types of cancer, different ICIs had manifested different cardiotoxicities. In melanomas, PD-1/PD-L1 inhibitor use was closely related to a fluctuation in blood pressure. Treatment-related hypertension was linked to lambrolizumab. Nivolumab appeared to have a correlation with ICIs-related hypotension. Abnormal blood pressures might also be caused by the toxic effect of ICIs on other organs (e.g., vasculature). In addition, fatal myocarditis was reported after a single treatment with the combination of nivolumab and ipilimumab [75]. Recent evidence suggests that abatacept, a CTLA-4 agonist, may be used as additional immunosuppression for severe ICI–related myocarditis [76]. In lung cancer, the common cardiotoxic manifestations of durvalumab were acute coronary syndrome, arrhythmia, and heart failure. The common cardiotoxic manifestations of nivolumab and pembrolizumab were arrhythmia, cardiac-related chest pain, cardiomyopathy, myopericardial disease, and pericardial disease. In renal cell carcinomas, nivolumab combined with ipilimumab appeared to cause hypertension. In urothelial carcinoma, atezolizumab was related to hypertension and arrhythmia. (4) In melanomas, we observed that the growing incidence of CAEs correlated with increased dosage [24] and frequency [26] of an ICI application. Regarding the cardiotoxicity of an ICI monotherapy compared with a combination therapy, two studies had inconsistent conclusions [25,30]. In lung cancer, two studies showed contradictory conclusions on the relationship between the ICI dose and ICIs-related cardiotoxicity [37,43]. As different drugs are used for different cancer types, the dosage and therapeutic regimens can also influence toxicity. Therefore, our conclusions require further evidence to be confirmed.

**Figure 2:**
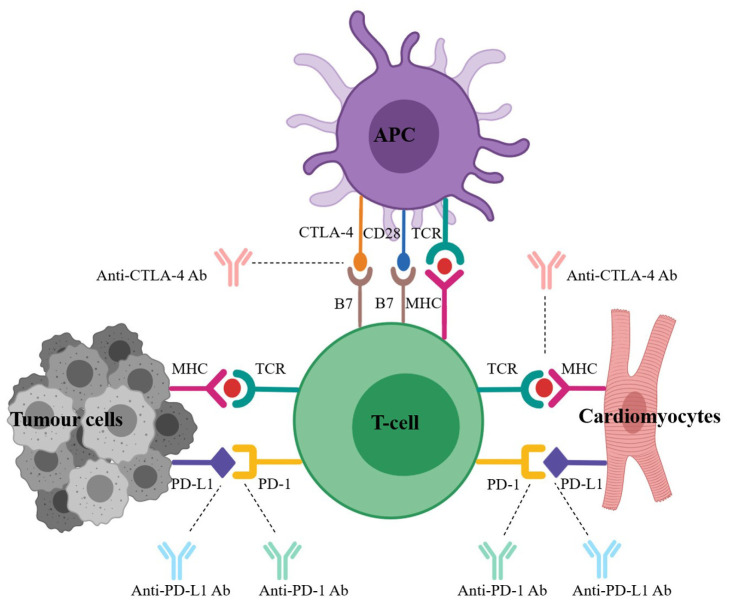
Tumor cells facilitate checkpoint activation to evade immune surveillance.

The pathogenic mechanism underlying ICIs-related cardiotoxicity has not been comprehensively studied [77]. Tumor cells escaping immune surveillance by promoting checkpoint activation have been recognized as a major mechanism (Figure 2). Direct T cell-mediated cytotoxicity leads to the inflammation of the His-Purkinje system. Furthermore, macrophage infiltration, inflammation, fibrosis of myocardium hyperactivation [78,79,80], infiltration of cytotoxic T cells into myocardial tissue, inhibition of cardioprotective PD-1 and PD-L1 pathways in cardiomyocytes, and clonal expansion of T cells against homologous tumors and myocardium antigens have been observed (Figure 2) [75,81]. Other hypotheses that have attracted attention are ICIs-associated inflammation-triggering destabilization [82,83,84], cytotoxic T cell activation leading to the pseudo-progression of pericardial micro-metastases [85,86,87,88], and direct action on the coronary vascular bed [89,90,91].

Tumor-intrinsic factors (such as a tumor-associated stroma) [92], patient-intrinsic factors, and environmental factors may be implicated in different cardiotoxicities of ICIs of different cancer types [93]. Tumor-intrinsic factors relating to the genetic, transcriptional, or functional profile of the tumor cells themselves [92,94] appear to be the decisive factors for ICIs-related cardiotoxicity. Patients with tumors having parallel histological and genetic features had a similar incidence of ICIs-related CAEs [92,95]. Tumor-intrinsic factors partook of the tumor-extrinsic mechanisms of ICIs-related cardiotoxicity through their effect on the interaction between the host immune system and the tumor [92,96]. The interval of time required for cardiotoxicity to occur has not yet been precisely indicated [97,98], so further work is required to elucidate this. There are still many unanswered questions about the effect of patient-intrinsic factors on ICIs-related cardiotoxicity because the mechanisms differ, even in patients treated with the same agent.

With a wide range of ICI applications in anticancer therapy, there is growing recognition of a broad spectrum of ICIs-related CAEs. More attention must be paid to cancer-type-specific ICIs-related cardiotoxicity to target high-risk patients so that effective prevention and treatment measures can be applied. For patients treated with ICIs, clinical management—including the observation of clinical symptoms, the detection of cardiac biomarkers, and the performance of electrocardiograms and echocardiograms—are strongly suggested. More importantly, cancer-type-specific clinical management is urgently required. In patients with NSCLC, we suggest that the dynamic monitoring of electrocardiograms be performed after ICI application to evaluate the occurrence of arrhythmias such as atrial fibrillation, conduction blocks, and even malignant arrhythmias. Regarding patients with cancers such as melanomas, renal cell carcinomas, and uroepithelial carcinomas, we suggest that blood pressure be monitored dynamically during ICI therapy.

For ICIs-related cardiac complications, a high dose of steroids a common treatment; however, there are some circumstances in which aggressive therapy may be ineffective [99,100,101]. According to ASCO guidelines, permanent discontinuation of ICIs is recommended for grade 4 toxicities, except for endocrinopathies that have been controlled by hormone replacement [102]. It is prudent for cardiologists and oncologists to spread awareness about the manifestations of ICIs-related cardiotoxicity for each cancer type and cooperate closely for its successful diagnosis and management. Rigorous follow-ups of patients receiving ICI therapy with cardiac biomarkers, EKGs, and echocardiograms are recommended. It should be borne in mind that different drugs are used for different cancer types, and if a drug causes a different toxicity in a particular cancer type, the composition of each drug should be compared. The dosage and therapeutic regimen should also be compared because they influence toxicity. Further studies focusing on exploring cancer-type-specific ICIs-related cardiotoxic manifestations and potential mechanisms are required and helpful for maintaining the cardiac health of cancer patients treated by chemotherapy.

## Data Availability

All data can be found in the references.
